# Cost-Effectiveness of a Community Exercise and Nutrition Program for Older Adults: *Texercise Select*

**DOI:** 10.3390/ijerph14050545

**Published:** 2017-05-20

**Authors:** Olufolake (Odufuwa) Akanni, Matthew Lee Smith, Marcia G. Ory

**Affiliations:** 1 Department of Economics, University of New Mexico, Albuquerque, NM 87131, USA; folakeodufuwa@gmail.com; 2 Institute of Gerontology, College of Public Health, The University of Georgia, Athens, GA 30606, USA; health@uga.edu; 3 Center for Population Health and Aging, Texas A&M School of Public Health, College Station, TX 77842, USA

**Keywords:** cost-effectiveness, program dissemination, physical activity, nutrition, Timed Up-and-Go, healthy days, EuroQol

## Abstract

The wide-spread dissemination of evidence-based programs that can improve health outcomes among older populations often requires an understanding of factors influencing community adoption of such programs. One such program is *Texercise Select*, a community-based health promotion program previously shown to improve functional health, physical activity, nutritional habits and quality of the life among older adults. This paper assesses the cost-effectiveness of *Texercise Select* in the context of supportive environments to facilitate its delivery and statewide sustainability. Participants were surveyed using self-reported instruments distributed at program baseline and conclusion. Program costs were based on actual direct costs of program implementation and included costs of recruitment and outreach, personnel costs and participant incentives. Program effectiveness was measured using quality-adjusted life year (QALY) gained, as well as health outcomes, such as healthy days, weekly physical activity and Timed Up-and-Go (TUG) test scores. Preference-based EuroQol (EQ-5D) scores were estimated from the number of healthy days reported by participants and converted into QALYs. There was a significant increase in the number of healthy days (*p* < 0.05) over the 12-week program. Cost-effectiveness ratios ranged from $1374 to $1452 per QALY gained. The reported cost-effective ratios are well within the common cost-effectiveness threshold of $50,000 for a gained QALY. Some sociodemographic differences were also observed in program impact and cost. Non-Hispanic whites experienced significant improvements in healthy days from baseline to the follow-up period and had higher cost-effectiveness ratios. Results indicate that the *Texercise Select* program is a cost-effective strategy for increasing physical activity and improving healthy dietary practices among older adults as compared to similar health promotion interventions. In line with the significant improvement in healthy days, physical activity and nutrition-related outcomes among participants, this study supports the use of *Texercise Select* as an intervention with substantial health and cost benefits.

## 1. Introduction

There is a greater recognition of social and environmental determinants of lifestyle factors, which play a critical role in morbidity and mortality across the life-course [1,2,3]. Important factors to be considered when assessing health problems and health outcomes of older adults in the community are physical activity and good dietary practices. Both have been associated with prevention or delayed onset of health conditions such as stroke, high blood pressure, coronary heart disease, type 2 diabetes, depression, some cancers, functional limitations, higher risk of falling, reduced cognitive function, reduced sleep quality and a reduced quality of life [4,5,6,7]. 

Regular physical activity is associated with lower mortality risk for older and younger adults because it decreases the risk of cardiovascular disease and some cancers [6,7,8,9]. For older adults, regular physical activity helps build stronger muscles to reduce the risk of falling and for continued independent living in the community [9,10]. Previous research has found a positive relationship between obesity/overweight from physical inactivity and functional limitations [11,12]. The economic burden or costs of physical inactivity have also been documented. Colditz [13] reported that economic costs from physical inactivity were approximately 2.4% of the United States (U.S.) healthcare expenditures in 1995 dollars. Ding and colleagues [14] also estimated the global economic cost of physical inactivity to be approximately $53.8 billion in 2013 on the healthcare system alone.

Economic costs from physical inactivity are usually classified into three categories: direct costs from medical care use; indirect costs from productivity loss and workers’ compensation; and forgone earnings from premature mortality attributed to physical inactivity [13,15]. Among the older adult population, most economic costs would accrue from direct medical care costs because a large percentage of older adults may be inactive and retired; hence, they may present limited or no productivity loss and forgone earnings.

Community-based physical activity interventions have been shown to help foster better health status, improve physical function, improve cognitive function, reduce risk of falling, improve quality of life, reduce symptoms of various illnesses and reduce healthcare costs [16,17,18,19,20,21]. However, despite these health benefits of physical activity in reducing morbidity and mortality and the cost savings from the reduced economic burden, the prevalence of leisure time physical activity among older adults is still lower than that of younger adults [9,22]. Possible racial and ethnic differences also exist as physical inactivity is reported to be more prevalent among blacks and Hispanics than non-Hispanic whites [23,24].

Research has also established the relationship between good nutritional habits and improved health. Several studies have related healthy dietary patterns to attenuate the decline in cognitive functioning that comes with the aging process [25,26]. For older adults, consuming a healthy variety of recommended foods is an important component of healthy nutrition and is a major recommendation of the 2010 dietary guidelines for Americans [27]. Racial and ethnic minorities have also been found to engage in less healthy dietary habits compared to whites [28,29].

National health organizations such as the U.S. Department of Health and Human Services (DHHS) and the U.S. Department of Agriculture (USDA) have proposed physical activity and dietary recommendations aimed at preventing or reducing mortality and improving overall quality of life [5,27]. Major barriers to participation in physical activity and healthy eating for the older population include lack of access to low-cost community-based programs [22], limited nutritional knowledge [30,31], low motivation [32], functional limitations, financial limitations and built-environmental limitations that could influence access to healthy food [33,34,35]. While a single strategy may not be able to reduce all aforementioned barriers, primary intervention strategies to improve both physical and nutrition lifestyles in the community include health education and health promotion programs, nutrition education campaigns and low-cost nutrition programs. One such program is the *Texercise Select*, a community-based program that follows the physical activity and nutrition recommendations of the DHHS and USDA to improve health outcomes among older adults. 

The primary goal of this cost-effectiveness study was to better understand contextual factors influencing the uptake of evidence-based health promotion programs. More specifically, the study objectives were to: (1) identify cost savings associated with health outcomes for *Texercise Select* participants; and (2) identify possible race/ethnicity-based disparities in program effectiveness and cost-effectiveness.

### The Texercise Select Program

*Texercise* is a health promotion and wellness program developed by the Texas Department of Aging and Disability Services (DADS) for older adults in the state of Texas [36,37]. The *Texercise Select* community program was an adaptation of the existing state’s *Texercise Classic* program [36]. It was developed with the primary goal of evaluating and establishing the evidence base for the on-going *Texercise* health promotion program in terms of reach and effectiveness. 

*Texercise Select* was administered at various locations in eight Texas counties selected based on contacts with the program developers and key clinical partners. Primarily located in Central Texas, the eight counties were a mix of rural and peri-urban counties. The selected counties were predominately comprised of non-Hispanic white residents; however, racial/ethnic populations were represented in each county (ranging from about 12 to 45% across the different counties).

*Texercise Select* was a 12-week program that included two weeks of program recruitment and 10 weeks of interactive classes. The objectives of the program were to improve participants’ knowledge about the value of physical activity and nutrition; increase participants’ confidence in their ability to make healthier choices related to physical activity and nutrition; improve participants’ mobility and increase the ease of sitting, standing and walking; and provide participants with effective strategies to prevent falling.

Twenty-nine facilitators were trained in a one-day six hour-long session [36]. A total of four facilitator trainings were conducted. Facilitators were recruited using existing community contacts with partners in the aging services and healthcare networks. In keeping with a lay leader model, there were no formal physical activity licensure requirements for volunteers to be trained as facilitators. Many of the trained facilitators for this study were previously trained as lay leaders for other evidence-based programs for older adults.

The cost of the program was minimized by holding the class sessions in local facilities, such as multi-purpose community facilities, senior centers, faith-based organizations and senior housing facilities. Recruitment was therefore influenced by attendance at these local facilities. These local facilities did not charge the Texas DADS for the use of their facilities. The first two weeks of the program were used for program recruitment, program presentation to participants and registration of participants, while the 10 weeks of classes included twenty 1.5-h workshops delivered in the various local facilities. Participants were recruited through a variety of communication channels regularly used by organizations in the aging services sector such as community presentations, flyers and word of mouth.

Classes were primarily marketed to adults aged 55 years and older (although those aged 45 and older were allowed to participate), and there was no maximum age for participation. The dates and times of the *Texercise Select* classes were advertised at each respective delivery site; participants who were interested were encouraged to sign up (i.e., by contacting the research team’s project coordinator or the point-of-contact identified at the delivery site). There were no physical or cognitive exclusionary criteria for participation, other than the participant needed to be capable of completing baseline and follow-up instruments. Encouraging engagement in appropriate physical activity to minimize exclusion of older adults who could benefit from the program, the Exercise Assessment and Screening for You (EASY) tool was used to document existing health conditions and identify immediate safety risks [38]. A safety tip sheet was also distributed to participants at the onset of the intervention.

Each session was structured to provide a variety of activities that were expected to help participants develop healthy behavioral skills in both physical activity and nutrition. For physical activity, each session included a 30–45 min exercise component that focused on building endurance, strength, balance and flexibility [36]. For dietary habits, sessions focused on teaching participants about healthy eating practices, such as incorporation of fruits and vegetables, portion control and healthy cooking. Participants were encouraged to complete daily physical activity and nutrition logs for the first four weeks of the program and set weekly physical activity and nutrition action goals. Individual progress on these action goals was reported at the beginning of each week, and possible barriers to making progress on the action goals were identified with helpful suggestions made by facilitators [36]. Each session also included interactive group activities such as group discussions or brainstorming centered on a specific health topic. These activities are expected to help participants develop the knowledge, skills and confidence to resume or increase physical activities and improve nutritional habits crucial to a healthy lifestyle.

Studies of *Texercise Select* have documented the effectiveness of the intervention to improve participants’ physical activity and nutrition habits and confidence [39], physical functioning [40] and self-reported quality of life [40].

## 2. Materials and Methods

### 2.1. Study Participants and Procedures

Program participants were surveyed using self-reported instruments distributed at each workshop location. Facilitators provided assistance to participants that needed help filling out the questionnaires. Data were collected at baseline (i.e., the first week of interactive classes) and follow-up (i.e., 10 weeks later upon the program’s conclusion). With the exception of sociodemographic information collected at baseline only, identical instruments were distributed at baseline and at follow-up. A total of 220 participants were registered at baseline, with 132 completing the follow-up assessment (60% data completion rate). This project received Institutional Review Board (IRB) approval through Texas A&M University (#IRB2013-0778D).

### 2.2. Data and Measures

*Sociodemographics*: Participant characteristics assessed in this study included the participant’s gender, age, race/ethnicity and level of educational attainment.

*Health indicators*: The Center for Disease Control (CDC) created four core measures to assess health-related quality of life (HRQOL) [41]. Specific wording and scoring for these self-reported HRQOL items are as follows:

- Item 1: Would you say that in general your health is excellent, very good, good, fair or poor? Scored on a scale of 1 to 5, with higher numbers representing worse health. Scores were reverse coded for this study as follows (1 = poor, 2 = fair, 3 = good, 4 = very good, 5 = excellent).
- Item 2: Thinking about your physical health, which includes physical illness and injury, how many days during the past 30 days was your physical health not good? Scored open-endedly, with responses ranging from 0 day to 30 days.
- Item 3: Thinking about your mental health, which includes stress, depression, and problems with emotions, how many days during the past 30 days was your mental health not good? Scored open-endedly, with responses ranging from 0 day to 30 days.
- Item 4: During the past month, how many days did poor physical or mental health keep you from doing your usual activities, such as self-care, work or recreation? Scored open-endedly, with responses ranging from 0 day to 30 days.

The summary index of unhealthy days was calculated by estimating the total number of days during the past month when the participant reported his or her physical or mental health as not good (that is, responses to the second and third questions above were summed, with a maximum cap of 30 days). The healthy days index was then derived by subtracting the number of unhealthy days from 30 days.

The *Timed Up-and-Go* (TUG) test is a measure of the time it takes an individual to rise up from an arm chair, walk three meters, turn, walk back to the chair and sit down again [42]. Participants are timed in seconds when performing the TUG activity. A faster time indicates better functional performance. Consistent with previous research, TUG scores of 12 or more seconds were used as a cut-off to identify older adults at increased risk of falls [40,43].

Participants reported the number of chronic health conditions they had ever been diagnosed with. Chronic illnesses assessed include diabetes, asthma, chronic obstructive pulmonary disease, high blood pressure, heart disease, cancer, arthritis and other lung conditions. 

*Physical activity*: Respondents were provided a standard set of description and examples of light (e.g., walking leisurely), moderate (e.g., brisk walking) and vigorous physical activity (e.g., jogging) after which they were assessed on their level of each type of physical activity based on the CDC’s recommended physical activity [5,44]. The specific physical activity question analyzed in this study was: Over the past 7 days, how many days did you do moderate to vigorous physical activity? Scores were self-reported and ranged from 0 day to 7 days.

*Nutritional habits*: Participants were asked questions assessing their fast food consumption, fruit/vegetable consumption and soda/sweetened drinks consumption based on the CDC’s recommendations for older adults [39]. The specific questions and response options were:

- Over the past 7 days, how many servings of fruits/vegetables did you eat each day? Scores were self-reported and ranged from 0 to 5 or more servings.
- Over the past 7 days, how many times did you eat fast food meals or snacks? Scores were self-reported and ranged from 0 to 5 or more days.
- Over the past 7 days, how many soda or sugar-sweetened drinks did you drink each day? Scores were self-reported and ranged from 0 to 5 or more drinks.
- In the average day, how many cups of water did you drink each day? Scores were self-reported and ranged from 0 to 8+ cups of water.

#### 2.2.1. Cost Measures

The measurement of costs used in this analysis was based on the actual direct costs of implementing the *Texercise Select* program. The total direct cost to deliver the 10-week physical activity and nutrition intervention was $50,474, which corresponded to an average cost of $229 per participant. Where applicable, costs were calculated based on an average of 19 enrolled participants per class (total number of participants = 220). For sensitivity analysis, costs were calculated using the maximum (*n* = 25) and minimum (*n* = 4) class size. Because the costs of the program incentives, personnel and recruitment were incurred up-front, per participant costs were calculated based on class enrollment, not class completion. Each cost category is described below:

*Cost of program incentive*: To encourage program participation, incentives were given to all participants during the program. These incentives include pedometers, handbooks, pledge sheets, resistance bands, t-shirts and certificates. The average price of all incentives was $6.91 per participant (equivalent to $131.29 per workshop class and a total program cost of $1520.20).

*Cost of recruitment and outreach materials*: During the program, all facilitators were given a kit containing recruitment materials at the beginning of the *Texercise Select* program. One kit was provided per class. The average cost of the kit was $8.19 per workshop class, which is equivalent to a cost of $0.43 per participant (and a total program cost of $94.60).

*Personnel cost*: Facilitators’ cost is calculated as hours worked multiplied by the cost per hour. Twenty-nine individuals were trained to facilitate the intervention program. The total estimated hours worked by each facilitator was 72 h. This includes the time spent on program awareness, recruitment of participants, planning, preparing and conducting each class session. The first two weeks of the 12-week program were assigned to program recruitment, program presentation and registration of participants. A one-day, six-hour training was conducted for facilitators. Recruitment time was 3 h per week (equivalent to a total of 6 h for two weeks). Estimated time for preparing for each class was 1.5 h per session (equivalent to a total of 30 h for 20 class sessions). Class preparation time takes into account the time spent setting-up/tearing down workshop materials and time spent on questions and answers after each session. Finally, during the 10 weeks, sessions were held twice a week for 90 min each (equivalent to 30 h for 10 weeks).

However, since all facilitators were voluntary participants with no expected monetary earnings from participation, the value of volunteer time was therefore calculated using the independent sector value of volunteer time. This value of volunteer time is the average wage of non-management, non-agricultural workers for Texas, extracted from the Bureau of Labor Statistics by the Independent Sector [45]. It is updated annually to reflect current price indices. It should be noted that the value of volunteer time is based on the volunteer work and not on the volunteer’s actual earning power or specialized skill. The independent sector’s value of volunteer time in Texas in 2013 was $23.40. Total estimated cost for each facilitator was therefore $1684.80 and a total cost of $48,859.20 for all 29 facilitators. This is equivalent to a per-participant personnel cost of $222.09. Sensitivity analysis is also conducted by using the average hourly wage rate of community and social service occupational group for the Bryan-College Station area to calculate the value of volunteer time, $20.01 in May 2013 [46].

Cost of participant time and travel cost of facilitators were excluded from cost analyses. The cost of participant time is usually measured by estimating the opportunity cost of participating in the program. Such costs can include forgone wages and value of leisure time. However, these are excluded because this study assumes the value of forgone wages will tend towards zero given the older age group of the participants. In other words, the participants may otherwise have been inactive and retired. The value of leisure time is also excluded given the difficulty in estimating such time for this age-group. This analysis also excludes the travel cost of facilitators, as it was not available, and it also was assumed to be minimal. In addition, this estimated cost also ignores economies of scale that could result from trained facilitators training other facilitators, as no evidence of that existed.

Costs are not discounted because of the short time horizon of the program. Costs are generally discounted in studies with a time horizon longer than one year [47].

#### 2.2.2. Outcome Measures

This study focused on two categories of outcomes for cost-effectiveness calculations: quality-adjusted life year (QALY) and selected physical activity- and health-related outcomes:

QALY outcome: The conventional and commonly-used QALY in cost-effectiveness analyses combines gains from reduced morbidity and mortality into a single measure ranging from 0 to 1, where a weight of 1 corresponds to perfect health and a weight of 0 corresponds to a state of health equivalent to death [48,49]. QALY is expressed in terms of “years lived in perfect health”. In other words, it is assumed that a year of life lived in perfect health is worth 1 QALY, and a year of life lived in a state of less than this perfect health is worth less than 1 (for example, 0.5 QALYs indicates half a year lived in perfect health). The number of QALY gain from a program is estimated by multiplying the preference based or utility values induced by the program by the duration of the program.

In contrast to the non-preference based CDC healthy days measure included in the *Texercise Select* assessment, QALYs can be calculated from a preference based HRQOL measure, such as the EuroQol 5D (EQ-5D) scores [50]. The EQ-5D is a preference-based measure of health status consisting of a descriptive system and visual analogue scale. Cost-effectiveness analyses focuses on the descriptive system, which assesses health in five categories—mobility, self-care, usual activities, pain/discomfort and anxiety/depression. Each category is assessed with 3 levels—no problem, some problems and severe programs. The descriptive system health measures are converted to a single summary index by attaching weights to each level in each category and deducting the weights from 1, the value for full health [51].

The non-preference-based healthy days measures utilized in this study are therefore converted to preference-based EQ-5D scores using a methodology proposed by Jia and Lubetkin [52], the HRQOL was converted into EQ-5D scores for the purposes of this study. The resultant EQ-5D scores were used to calculate QALYs using the area under the curve approach, where the total study period is divided into time intervals corresponding to the number of follow-up assessments, and each interval is weighed by the individual’s utility (EQ-5D) scores during that period of time [53]. With the assumption that the individuals’ utility was neither missing nor censored, QALYs can be calculated as shown below:

(1) $$Incremental\;QALYs=\;\overset{n}{\underset{t=1}{\sum }}\frac{Q_{t}+Q_{t+1}}{t+1}.\;\;D_{t}$$

*Q_t_* represents the individuals’ EQ-5D scores during period *t* (at baseline) and *D_t_* is the time duration for period *t* (=2.5 months in this study) usually expressed as a fraction of twelve months [53]. *n* represents the number of time intervals or follow-ups (=1 in this study). Average EQ-5D scores for all participants in this study are substituted for *Q_t_* and *Q*_(*t*+1)_ respectively. For example, for a respondent with estimated EQ-5D score at baseline and post-intervention of 0.811 and 0.883, respectively:

(2) $$Incremental\;QALY=\left[\;\frac{0.811+0.883}{2}\;.\;\frac{2.5}{12}\;\right]=0.176$$

*Physical activity and health-related outcomes*: Program effectiveness was also measured with three physical activity- and health-related outcomes. The first outcome was the number of users who reported an improvement in healthy days. The second was the number of users who reported an increase in the days engaged in moderate to vigorous physical activity each week. This outcome indicates the effectiveness of the program in improving knowledge about and promoting physical activity. The third outcome was the number of participants who reported an improvement in TUG test scores. All three outcomes collectively accessed quality of life among participants from baseline to follow-up.

### 2.3. Statistical Methods

Cost-effectiveness deals with comparing the difference in costs and outcomes between interventions through the calculation of an incremental cost-effectiveness ratio (ICER). The ICER compares the difference in costs between two mutually-exclusive interventions to the difference in effectiveness between the interventions as shown in the equation below. In the current analyses, we are comparing *Texercise Select* (intervention) to a no intervention (zero) control.

(3) $$ICER=\frac{C_{i}-C_{0}}{E_{1}-E_{0}}$$

Here, *C_i_* represents the cost of the program of the study, while *C*_0_ is the cost of the default program. In this study, the default alternative is a do-nothing or no-intervention option; hence, there is zero average cost for the alternative option. In this default alternative, participants are assumed to go about their daily activities and nutrition routine like they were before the program implementation. In other words, *C*_0_ and *E*_0_ are assumed to be zero, indicating that the numerator represents the average cost for implementing the *Texercise Select*, and the denominator represents the specific outcome measure.

Similarly, *E*_1_ and *E*_0_ represent the effectiveness of the program of study and the default program, respectively. The default program is usually the next best alternative. The ICER can also be described as the ratio of incremental costs to incremental outcomes (say, QALY). The incremental cost is the difference between the average cost of the intervention and the average cost with no intervention. Similarly, incremental QALYs represent the difference between the gained QALY from the program and the gained QALY with no intervention [54,55]. ICER is the most widely-used technique for cost-effectiveness analyses.

This cost-effectiveness analysis is conducted from a societal perspective with all relevant costs and effects being measured. Program effectiveness is assessed using QALY, healthy days, days engaged in physical activity each week and TUG test scores. 

Cost-effectiveness analysis with QALY outcome: The cost-effectiveness ratio with QALY outcome of the *Texercise Select*, relative to no intervention alternative, was calculated by dividing the average cost by the average QALYs gained as shown below. The ratio indicates the cost per QALY gain.

(4) $$ICER=\;\frac{Average\;cost\;per\;participant\;-\$0}{QALYs\;gained\;-0}$$

*Cost-effectiveness analysis with selected health outcomes*: Here, cost-effectiveness ratios were calculated by dividing the average cost by each non-monetary unit of the three selected physical activity and health-related outcomes. For the healthy days outcome, the ratio indicates the cost required for one individual who reported an improvement in healthy days. For the physical activity outcome, the ratio indicates the cost required for an individual who reported an increase in days engaged in moderate to vigorous physical activity each week. Finally, the cost-effectiveness ratio with the TUG outcome indicates the cost required for one individual who reported an improvement in TUG scores as a result of the program.

(5) $$ICER=\;\frac{Average\;cost\;per\;participant\;-\$0}{Units\;of\;outcome\;-0}$$

## 3. Results

*Participant characteristics*: Baseline and follow-up sample characteristics of the respondents are presented in Table 1. Analyses were based on matched baseline and follow-up assessments. The average age of the respondents was 75 years. Females (84%) and non-Hispanic whites (79%) constituted the largest percentage of the respondents. Eight percent of the respondents were Hispanic, and 13% were black. Forty percent of the sample was married. The average number of chronic conditions and self-rated health reported at baseline was 2.40 and 3.02, respectively. High blood pressure and arthritis were the most common conditions reported by the participants, with 73% and 64% of participants reporting each condition, respectively.

*Program health and behavioral effects*: Follow-up statistics are presented for select time-invariant variables. There was a significant increase in self-rated health from 3.02 to 3.28 at follow-up, indicating that respondents rated health higher at the completion of the program. TUG scores significantly decreased from 13.03 s to 11.53 s at follow-up, indicating improved functional performance at program conclusion. Physical activity days in a week significantly increased from 2.8 to 4.0 days at follow-up. Fruits/vegetable servings in a week significantly increased from 3.3 to 3.7 servings, while daily cups of water consumed significantly increased from 5.5 to 6.03 cups.

*HRQOL outcomes*: Descriptive statistics of the HRQOL measures for the overall sample are presented in Table 2. Respondents reported approximately 20 healthy days at baseline, and the estimated average EQ-5D score was 0.75 at baseline. Follow-up statistics reveal a significant increase in the HRQOL at program follow-up (increase to 23 healthy days). Higher EQ-5D values correspond to a better health state, thus indicating the positive impact of the *Texercise Select* in improving health. There was improvement in the EQ-5D level, with significance at the *p* < 0.1 level.

*Correlates between HRQOL and socio-demographic characteristics*: Table 3 presents summary statistics for healthy days and corresponding EQ-5D scores by socio-demographic characteristics at baseline and follow-up. Differences also existed in EQ-5D scores by socio-demographic characteristics, both at baseline and follow-up. On average, females reported more healthy days and had higher EQ-5D scores compared to males, both at baseline and follow-up. For racial and ethnic categories, Hispanics reported more healthy days at baseline, yet they did not report improvements from baseline to follow-up. Additionally, some improvements were observed for both healthy days and EQ-5D scores among non-Hispanic whites and blacks, but only non-Hispanic whites experienced significant (*p* < 0.05) improvements in healthy days from baseline to the follow-up period.

Cost analyses: Table 4 details the cost analyses of the *Texercise Select* program. The average cost per participant was $229, while the total program cost for all participants in all counties was $50,474. 

Cost-effectiveness ratios for the *Texercise Select* program are presented in Table 5. The average QALY gain of 0.159 for the overall population resulted in an incremental cost per QALY gain of $1443. This ratio is lower when compared to the common cost-effectiveness threshold of $50,000 for a gained QALY and also in comparison to other health promotion interventions. When comparing cost per QALY across racial and ethnic groups, the average QALY gain of 0.158, 0.167 and 0.160 for non-Hispanic whites, blacks and Hispanics resulted in a cost per QALY gain for $1452, $1374 and $1434, respectively. Thus, in comparison to an alternative strategy of no program, a physical activity and nutrition program such as the *Texercise Select* will require an investment ranging from $1374 to $1452 for each QALY gain. 

Cost-effectiveness ratios with the healthy days outcome ranged from $6 to $76 to achieve each individual improvement. Similarly, cost-effectiveness ratios with the weekly physical activity and TUG outcomes ranged from $3 to $57 and $7 to $76, respectively. Results revealed that it will cost more to achieve each individual improvement in healthy days, days of physical activity each week and TUG scores for Hispanics and blacks, compared to non-Hispanic whites. However, this could be attributed to the small number of Hispanics and blacks in the final sample. Non-Hispanic whites constituted the largest percentage of the population (79%).

To determine the robustness of the final results, sensitivity analyses were conducted by varying the workshop class size and calculating the value of volunteer time with the average hourly wage rate of the community and social service occupational group for the intervention site area. The class size of the program is varied by using the maximum class size of 25 participants and the lowest class size of four participants. Results are consistent across all parameter variations as cost-effectiveness ratios are comparable to the initial ratio. Using the maximum class size lowered the ICER to $1442, while using the minimum class size increased the ICER to $1453 per QALY gained. Finally, re-estimating the value of volunteer time lowered the ICER to $1241 per QALY gained.

## 4. Discussion

This study marks the first attempt to evaluate the cost-effectiveness of the *Texercise Select* program, shedding light on important considerations for program dissemination and sustainability. In particular, it adds to the scant literature about the cost-effectiveness of heath interventions for older adults [56]. Program effectiveness was measured using QALY gained, as well as health outcomes such as healthy days, physical activity and Timed Up-and-Go (TUG) test scores. Preference-based (EQ-5D) scores are estimated from the number of healthy days reported by participants and converted into QALYs. Additionally, sociodemographic differences in the program and cost-effectiveness were also analyzed.

The average cost of the intervention per participant was $229. This cost appears to be inexpensive compared to similar short time-horizon interventions to improve physical activity and prevent falls among older population. Timonen et al. [57] reported an average cost of 568 EUR (corresponding to $636) per participant for a 10-week group-based exercise program to improve physical fitness and functional abilities in frail elderly women after discharge from hospital. Similarly, Rizzo et al. [58] reported an average cost of $905 per participant for a fall-prevention program for an older community population. It should also be noted that volunteer cost constituted over 96% of the total program cost. Thus, policy-makers and health agencies considering the implementation of the *Texercise Select* program may not have to pay the volunteer portion of the total cost out-of-pocket.

Results reveal the *Texercise Select* program to be cost-effective as the cost-effectiveness ratio ranged from $1374 to $1452 per QALY gain, which is much lower when compared to the common cost-effectiveness threshold of $50,000 and also in comparison to other health promotion interventions. Munro et al. [59] reported an incremental cost per QALY of £17,174 (corresponding to $26,373) for a community-based exercise program for the older population. Eriksson et al. [60] estimated a cost per QALY gain ranging from $1668 to $4813 of a health intervention that consisted of similar group-based physical activity trainings and nutrition counselling. Additionally, some improvements were observed for both healthy days and EQ-5D scores among non-Hispanic whites and blacks, even though only non-Hispanic whites experienced significant improvements in healthy days from baseline to the follow-up period. However, the low socio-demographic impacts could be attributed to the small number of Hispanics and blacks in the sample.

Given the cost-effectiveness and the positive health impacts of the program, this study recommends an expansion of the program (both within the study area and beyond). However, some possible amendments may be helpful to ensure that observed positive changes in individual physical activity and nutrition choices are sustained over time and to help ensure a more efficient future economic analysis such as longer follow-up time periods. For example, rather than a single follow-up at the end of the program, a detailed follow-up could also be conducted on all participants at six-month and twelve-month periods after program completion. 

This study helps debunk the myths that older adults are incapable of benefiting from engaging in regular exercise or physical activity [61]. Further, analyses on other physical activity and nutrition indicators at follow-up compared to baseline indicated positive effects from the program as participants showed an increase in days of moderate to vigorous physical activity physical activity, increased weekly fruit/vegetable intake and increased daily water intake [39,40]. Overall, the positive outcomes from the *Texercise Select* could indicate that physical activity and nutrition-related interventions may be more beneficial if they are supervised in an organized setting or if a support system is available. 

Developing health promotion programs for older adults has raised concerns because of the perception that older adults may have difficulties following the physical activity and nutrition lifestyle changes after the intervention program ceases [4]. Yet, older adults may benefit from continuous collaborative partnership between the individual and a support system (formal or informal) to ensure adherence to healthy lifestyle changes or plans. Thus, to enhance attendance rates and promote ongoing positive lifestyle changes, a strategy for participation and outcome may be for participants to “buddy up” with other class participants and form naturally-occurring support groups for continued engagement in group activities and support for any identified barriers (e.g., transportation to group-based activities). The action planning and goal setting learned within class should be practiced and reinforced after the class ends, perhaps through reunion events where participants and facilitators are invited to report on successes and problem solve any lingering changes to engaging in healthy lifestyles. Engaged participants might also consider being trained to become lay facilitators, spreading program benefits to others, as well as themselves. 

Another beneficial amendment to the *Texercise Select* program is creating a control group in addition to the intervention group, where respondents in the control groups are provided general written and/or verbal information on required exercise and nutritional habits at baseline with no further on-site workshops or supervision. This could help estimate a better and more detailed cost-effectiveness analysis where cost-effectiveness ratios are estimated for the intervention group and compared with the control group. Pre- and post-program program instruments could also be improved by including preference-based health measures in the assessment instrument, such as the EQ-5D health states. This will help eliminate any possible uncertainties from converting the non-preference CDC healthy days measure to preference-based EQ-5D measures, in trying to calculate QALYs. Ideally, participants’ costly healthcare utilization (e.g., emergency room visits and hospitalizations) could be monitored over time.

This cost-effectiveness study is not without its limitations. Some observed outcomes may be the result of other programs or events other than the one being analyzed. In addition, this analysis could be underestimating the total costs of the program because healthcare costs that might be avoided were not included in cost calculations. Additionally, estimates of disease incidence avoided are not included. Both estimates were excluded due to the short time frame of the program. Because per participant costs were calculated based on class enrollment and not class completion, per participant costs would be considerably higher if calculated for only those who completed the class. Future studies should examine the per participant costs associated with the intervention dose received by participants. 

This study took a parsimonious and conservative approach by assuming that the short time period of the *Texercise Select* program may not result in any significant savings in healthcare costs or a reduction in disease incidence. Any cost underestimation, if any, will therefore be minimal and may not have a significant influence on these cost-effectiveness results. 

In addition, the generalizability of the findings may be limited due to the small sample size especially among Hispanics and blacks and study attrition. Some participants appeared to treat the program as a “drop in program” and therefore missed key baseline or follow-up data. Further analysis of attrition also revealed that respondents with fewer self-reported healthy days were less likely to have both baseline and follow-up data. No other significant differences existed between those with complete data assessments versus those without both baseline and follow-up data. 

This finding may indicate a healthful participant bias, where those who are sicker are unable to regularly attend class sessions (e.g., because of barriers not experienced by healthier participants including functional limitations or more frequent visits to the doctor). Thus, additional analyses are warranted to examine factors associated with *Texercise Select* attrition, which are not possible given the small number of racial/ethnic participants. For example, future studies should examine the intersection of health status and race/ethnicity to identify whether subgroups are disproportionately burdened by health problems that impact program attendance.

## 5. Conclusions

This study is one of the first cost-effectiveness analyses of an intervention focused on improving both physical activity and nutritional habits among the older population in Texas. Basic cost-effectiveness analyses are conducted by comparing the actual direct costs of the *Texercise Select* program to the QALY gain and to other outcomes, the estimated number of respondents who reported an improvement in healthy days, days of physical activity each week and in TUG scores. 

The significant health benefits from the program provide evidence of the benefits of physical activity and good dietary habits in older people. The increasing life expectancy and the shift towards an older population has increased the need for maintaining or improving the health of the older population [62].

This study helps provide an understanding of the financial resources needed to implement such a program and thereby help in achieving the CDC Healthy People 2020 objective of “reducing the proportion of older adults who have moderate to severe functional limitations” and “increasing the proportion of older adults with reduced physical or cognitive function who engage in leisure-time physical activities” [63]. It also provides evidence for the potential benefits that can be attributed to an evidence-based intervention in terms of cost-effectiveness ratios.

In the context of the ongoing movement to deliver evidence-based programs to older adults in the United States [21], health policy-makers are encouraged to recognize the potential of *Texercise Select* and similar programs to improve health and promote healthy habits among older adults. Economic evidence of successful interventions from evaluation studies such as this are capable of: (1) influencing funders to allocate resources for prevention rather than treatment; (2) garnering excitement within communities to build partnerships across organizations to leverage participants and funding; and (3) enhancing practice to efficiently reach and serve older adults to avert costly and preventable healthcare interactions.

## Figures and Tables

**Table 1:** Descriptive statistics: baseline and follow-up.

Variables	Baseline Mean (SD)	Follow-Up Mean (SD)	*p*
*Time-Invariant Variables*			
Age (in years)	74.70 (8.41)		
Female (%)	84.09		
Non-Hispanic white (%)	79.07		
Hispanic (%)	7.75		
Blacks (%)	13.18		
Married (%)	40.45		
Education	3.61 (1.09)		
Number of Chronic conditions	2.40 (1.46)		
*Time-Varying Variables*			
TUG	13.03 (5.19)	11.53 (4.38)	<0.01
Self-rated health (1 to 5)	3.02 (0.89)	3.28 (0.94)	<0.01
Physical activity days	2.79 (2.14)	3.96 (1.79)	<0.01
Fast food consumption	2.03 (1.62)	1.80 (1.55)	0.10
Fruits/Vegetables consumption	3.34 (1.42)	3.70 (1.24)	0.03
Soda consumption	1.06 (1.34)	0.98 (1.29)	0.45
Water consumption	5.49 (1.99)	6.03 (1.84)	<0.01

*N* = 132; Standard deviations (SD) in parentheses; TUG represents Timed Up-and-Go and EQ-5D represents EuroQol scores; Source: *Texercise Select* data 2013.

**Table 2:** Descriptive Statistics: health-related quality of life (HRQOL) measures.

HRQOL Measures	Baseline Mean (SD)	Follow-Up Mean (SD)	*p*
Healthy days (0 to 30)	20.27 (12.13)	22.71 (10.99)	0.02
EQ-5D (0 to 1)	0.75 (0.17)	0.77 (0.16)	0.09

*N* = 132; SD in parentheses; EQ-5D represents EuroQol scores; Source: *Texercise Select* data 2013.

**Table 3:** HRQOL measures by socio-demographic characteristics.

Variables	Healthy Days		*p*	EQ-5D		*p*
	Baseline	Follow-Up		Baseline	Follow-Up	
*Sex*						
Female	21.14 (11.57)	23.51 (10.24)	0.04	0.76 (0.17)	0.78 (0.14)	0.10
Male	15.67 (14.15)	18.48 (13.82)	0.13	0.70 (0.20)	0.72 (0.20)	0.51
*Race and Ethnicity*						
Non-Hispanic white	20.20 (12.22)	22.84 (11.02)	0.02	0.75 (0.17)	0.77 (0.15)	0.10
Hispanic	22.60 (10.10)	21.30 (11.68)	0.74	0.78 (0.13)	0.76 (0.17)	0.69
Blacks	21.35 (12.05)	25.12 (8.45)	0.19	0.78 (0.17)	0.82 (0.12)	0.21

*N* = 132; SD in parentheses; EQ-5D represents EuroQol scores; Source: *Texercise Select* data 2013.

**Table 4:** Costs.

	Per Participant ($)	Per Program ($)
Incentives	6.91	1520.20
Recruitment and outreach	0.43	94.60
Personnel	222.09	48,859.20
Total	229.43	50,474

Total program costs are calculated using a total participant number of 220.

**Table 5:** Cost-effectiveness ratios.

Category	Outcome	Cost-Effectiveness Ratio
*Overall:*		
QALY ^a^	0.159	1442.96
Healthy days (#)	50	4.59
Physical activity (#)	82	2.80
Timed Up-and-Go (#)	88	5.34
*Non-Hispanic White*		
QALY ^b^	0.158	1452.09
Healthy days (#)	39	5.88
Physical activity (#)	68	3.37
Timed Up-and-Go (#)	70	7.40
*Black*		
QALY ^c^	0.167	1373.83
Healthy days (#)	7	32.78
Physical activity (#)	9	25.49
Timed Up-and-Go (#)	9	28.68
*Hispanic*		
QALY ^d^	0.160	1433.94
Healthy days (#)	3	76.48
Physical activity (#)	4	57.36
Timed Up-and-Go (#)	7	76.48

QALY: quality adjusted life year; Cost-effectiveness ratio = average cost/outcome measure; Non-Hispanic whites *N* = 102, blacks *N* = 17 and Hispanics *N* = 10; # signifies the number of participants who reported an increase in the outcomes; ^a^ Incremental QALY overall = [(0.749 + 0.773)/2 * 2.5/12 = 0.159]; ^b^ Incremental QALY overall = [(0.747 + 0.773)/2 * 2.5/12 = 0.158]; ^c^ Incremental QALY overall = [(0.779 + 0.823)/2 * 2.5/12 = 0.167]; ^d^ Incremental QALY overall = [(0.775 + 0.757)/2 * 2.5/12 = 0.160].
